# Effectiveness of a multicomponent PDCA-based infusion safety management strategy in patients with pulmonary tuberculosis: a non-randomized historical-control study

**DOI:** 10.3389/fmed.2026.1899920

**Published:** 2026-10-06

**Authors:** Xiaoming Wang, Haiyan Fu, Zuohui Shu, Yaru Wang, Gege Ren, Yaru Li

**Affiliations:** 1 Department of Anesthesiology, The Fourth Hospital of Inner Mongolia Autonomous Region, Hohhot, China; 2 Department of Intensive Care Unit, The Fourth Hospital of Inner Mongolia Autonomous Region, Hohhot, China; 3 Department of Respiratory Medicine, The Fourth Hospital of Inner Mongolia Autonomous Region, Hohhot, China; 4 Department of Nursing, Inner Mongolia Medical University, Hohhot, China

**Keywords:** operating room, PDCA cycle, peripheral intravenous catheter, pulmonary tuberculosis, safety management

## Abstract

**Objective:**

To evaluate the association between a multicomponent PDCA-based infusion safety management strategy and intraoperative peripheral intravenous (PIV) outcomes in adults with pulmonary tuberculosis.

**Methods:**

This non-randomized historical-control study included 92 adults undergoing thoracic surgery: 46 historical controls receiving routine infusion management and 46 patients receiving a multicomponent intervention integrating PDCA-based quality improvement, staff training, standardized protocols, interdepartmental collaboration, audit and feedback, and two auxiliary devices. Baseline demographic and surgical characteristics were comparable between groups.

**Results:**

Compared with historical controls, the intervention group had lower rates of erythema/tenderness [8.7% vs. 69.6%; RR, 0.13 (95% CI, 0.05–0.33)], catheter dislodgement [2.2% vs. 28.3%; RR, 0.08 (95% CI, 0.01–0.56)], and catheter occlusion [4.3% vs. 43.5%; RR, 0.10 (95% CI, 0.02–0.40); all *P* ≤ 0.001]. Catheter dwell time was longer [5.0 vs. 3.0 days; Hodges–Lehmann difference, +2.0 days (95% CI, 1.0–2.0)], whereas hospital stay was shorter [11.0 vs. 19.0 days; difference, −7.0 days (95% CI, −10.0 to −5.0)]. Satisfaction scores were also higher [95.0 vs. 91.5; difference, +3.0 points (95% CI, 2.0–4.0); all *P* < 0.001].

**Conclusion:**

The multicomponent PDCA-based strategy was associated with fewer PIV-related safety events, longer catheter dwell time, and higher satisfaction. However, because the intervention combined process changes with two devices and used a historical control, the observed differences cannot be attributed independently to PDCA or either device.

## 1 Introduction

The role of surgical intervention in the management of tuberculosis (TB) in China has undergone a strategic transformation—from marginalization to precise clinical positioning (1–3). In the early era of anti-tuberculosis pharmacotherapy during the 20th century, surgical approaches were largely overlooked. However, with the rising prevalence of drug-resistant tuberculosis (DR-TB) and the persistent clinical challenges posed by complex forms such as spinal TB, the value of surgical treatment has been reappraised and reintegrated into the modern comprehensive TB control framework (4–6). Surgical management now plays an irreplaceable role in TB care and has rapidly evolved from traditional open procedures toward minimally invasive, precision techniques (7–9). This paradigm shift has significantly expanded the indications for surgical intervention while enhancing overall safety.

Intravenous infusion is a fundamental nursing practice and is particularly critical in the operating room setting. It spans the entire perioperative process and serves as a vital support for ensuring surgical success. Intravenous infusion is essential not only for intraoperative drug administration and symptom control but also for emergency resuscitation and the maintenance of water-electrolyte balance, acid-base homeostasis, and fluid volume stability. In clinical practice, however, inadequate attention to infusion lines, lack of operational proficiency, insufficient assessment, and limited knowledge of catheter care among nursing staff can result in unsafe infusion practices. Such issues may contribute to peripheral intravenous catheter failure and complications including infiltration, extravasation, occlusion, phlebitis, dislodgement, and infection, potentially interrupting treatment and compromising patient safety (10, 11).

Ensuring patency of intravenous access during surgery is crucial, as it serves as a key channel for emergency interventions. Consequently, enhancing the safety management of intravenous infusion in the operating room is of paramount importance. The PDCA (Plan–Do–Check–Act) cycle, introduced by American quality management expert W. Edwards Deming in the 1950s, emphasizes continuous quality improvement across all management activities through a structured iterative process (12). This scientifically grounded model incorporates four core components planning, implementation, evaluation, and adjustment and has demonstrated remarkable success in healthcare administration, particularly in improving perioperative nursing quality. Its application has led to measurable improvements in nursing outcomes and broader enhancements in medical service quality, with numerous studies confirming its practical efficacy (13).

Despite these advantages, the application of the PDCA cycle in the intraoperative intravenous infusion safety management of TB patients remains suboptimal. Two critical challenges contribute to this gap. First, peripheral intravenous catheters are susceptible to mechanical and local complications, including dislodgement, infiltration, occlusion, phlebitis, and premature catheter failure, particularly when catheter stabilization, surveillance, or handling is suboptimal. Second, perioperative intravenous access requires reliable catheter patency and securement because anesthetized or sedated patients may be unable to report early symptoms of infiltration or device-related complications. These considerations support the use of standardized vascular-access practices, structured catheter surveillance, and appropriate securement strategies as part of perioperative infusion safety management.

To address these limitations, the present study incorporated two proprietary devices—a regulated peripheral intravenous catheter stabilizer and an intraoperative intravenous drug delivery system into the PDCA management framework. These innovations were applied specifically to the safety management of peripheral venous infusions in the operating room. To date, no comparable research has been published internationally. Therefore, the findings of this study may offer valuable insights for enhancing infusion safety, improving patient comfort during surgical treatment, and potentially elevating the overall effectiveness of care in this domain.

## 2 Materials and methods

### 2.1 Study design and participants

This single-center, non-randomized historical-control study compared patients managed during two consecutive treatment periods at our institution. Adult patients with pulmonary TB who met the indications for thoracic surgery and were treated between June 2023 and April 2025 were eligible. Patients treated between June 2023 and June 2024 constituted the historical control cohort, whereas patients treated between July 2024 and April 2025 constituted the intervention cohort after implementation of the multicomponent infusion safety management strategy. Group allocation was therefore determined by treatment period rather than randomization. For the intervention cohort, written informed consent was obtained from all participants. For the historical control cohort, the Institutional Ethics Committee of The Fourth Hospital of Inner Mongolia Autonomous Region approved a waiver of informed consent because the study used de-identified archived medical records, and the waiver did not adversely affect patient rights or welfare (Approval No.: KY2024017). All study data were de-identified before analysis, and patient confidentiality was maintained in accordance with institutional requirements.

For study planning, the sample-size calculation was based on the expected between-group difference in a major PIV-related complication. Based on preliminary institutional data, the event rate was assumed to decrease from approximately 45% under conventional infusion management to 15% after implementation of the multicomponent strategy. With a two-sided *α* of 0.05% and 80% statistical power, approximately 36 patients per group were required for comparison of two independent proportions. Allowing for incomplete or unavailable records, the investigators aimed to include at least 40 patients per group. Ultimately, 46 patients were included in each cohort. The final study included 46 patients in each group, exceeding the minimum calculated sample size.

After determination of the minimum sample size, the historical control cohort was identified retrospectively from consecutive eligible patients who underwent surgery between June 2023 and June 2024 and received routine intravenous infusion management. The intervention cohort consisted of consecutive eligible patients treated between July 2024 and April 2025 after implementation of the multicomponent infusion safety management strategy. The same clinical eligibility criteria were applied to both cohorts to improve comparability between the two periods. No random allocation or concurrent control group was used.

#### 2.1.1 Intervention group inclusion and exclusion criteria

**Inclusion criteria**:

1. Age ≥18 years at the time of surgery;
2. Diagnosis consistent with the national criteria for pulmonary tuberculosis as defined in WS 288—2017;
3. Presence of drug-resistant TB and surgical indication due to complications;
4. Clear consciousness, normal cognition, and ability to communicate verbally;
5. Full understanding of the study, with written informed consent obtained as required by the approved study protocol.

**Exclusion criteria**:

1. History of psychiatric disorders, consciousness disturbances, chronic respiratory failure, pulmonary encephalopathy, or other severe complications;
2. Requirement for continuous noninvasive or invasive mechanical ventilation, or hemodynamic instability;
3. Dependence on enteral or parenteral nutrition;
4. Incomplete medical records.

#### 2.1.2 Control group inclusion and exclusion criteria

For the control group, eligible patients were retrospectively selected from the medical records of individuals who had previously received care for pulmonary tuberculosis at our institution.

**Inclusion criteria:**

1. Age ≥18 years at the time of surgery;
2. Diagnosis consistent with the national criteria for pulmonary tuberculosis (WS 288—2017);
3. Presence of drug-resistant tuberculosis and documented surgical indication due to complications;
4. Clear consciousness, normal cognition, and documented ability to communicate effectively;
5. Complete medical records permitting reliable assessment of the clinical course and study outcomes.

**Exclusion criteria:**

1. History of psychiatric disorders, consciousness disturbances, chronic respiratory failure, pulmonary encephalopathy, or other severe complications as recorded in the medical chart;
2. Requirement for continuous noninvasive or invasive mechanical ventilation, or hemodynamic instability at the time of hospitalization;
3. Dependence on enteral or parenteral nutrition;
4. Incomplete or missing medical records that precluded reliable evaluation of treatment processes or outcomes.

A total of 46 patients were ultimately included in both the control and intervention groups, meeting the minimum sample size requirement.

### 2.2 Intravenous infusion management

#### 2.2.1 Control group

Patients in the control group received routine intravenous infusion management according to standard nursing protocols. This included verification of patient and medication information, strict adherence to sterile puncture procedures, secure fixation of the intravenous catheter, regulation of infusion rate, and communication with family members regarding intravenous therapy and surgical precautions.

#### 2.2.2 Intervention group

In addition to routine intravenous infusion care, patients in the intervention period received a multicomponent infusion safety management strategy organized within the PDCA quality-improvement framework. The intervention comprised two conceptually distinct categories: (1) process-management components, including staff training, standardized infusion and catheter-management protocols, interdepartmental collaboration, routine audit, feedback, and iterative quality improvement; and (2) device-related components, consisting of a novel intraoperative intravenous drug-delivery device and an adjustable PIVC stabilizer. All components were introduced as an integrated care bundle during the intervention period. The specific measures were as follows:

#### 2.2.3 Plan (P): Needs assessment and goal setting

A baseline analysis was conducted to identify common issues related to peripheral intravenous catheter (PIVC) management:

1. absence of available venous access;
2. improper catheter placement (e.g., right limb or near joint areas);
3. inappropriate catheter size leading to poor flow;
4. poor fixation technique resulting in dressing edge peeling;
5. multiple scalp vein sets sharing one heparin cap;
6. improper handling during patient transfer leading to catheter occlusion;
7. backflow and blockage due to surgical staff oversight at the end of surgery.

Following discussions among the nursing team, and considering the strict aseptic requirements of the operating room as well as the difficulty of vascular access in some TB patients, the primary improvement goal was defined as increasing the “first-attempt success rate of intravenous cannulation.”

#### 2.2.4 Do (D): implementation of the multicomponent intervention

##### 2.2.4.1 Staff training and competency reinforcement

The head nurse or designated staff conducted regular ward rounds and structured training sessions to standardize intravenous infusion safety practices, catheter maintenance, and management during intra-hospital transport.

##### 2.2.4.2 Standardized clinical protocols

Standardized procedures were implemented for vessel selection, catheter selection, catheter flushing and sealing, tubing management, patient transport, and postoperative catheter management.

##### 2.2.4.3 Device-related components

Two newly introduced devices were used during the intervention period: a novel intraoperative intravenous drug-delivery device (Figure 1) and an adjustable PIVC stabilizer (Figure 2). These devices were intended to facilitate standardized drug administration and improve catheter stabilization, respectively.

**Figure 1:**
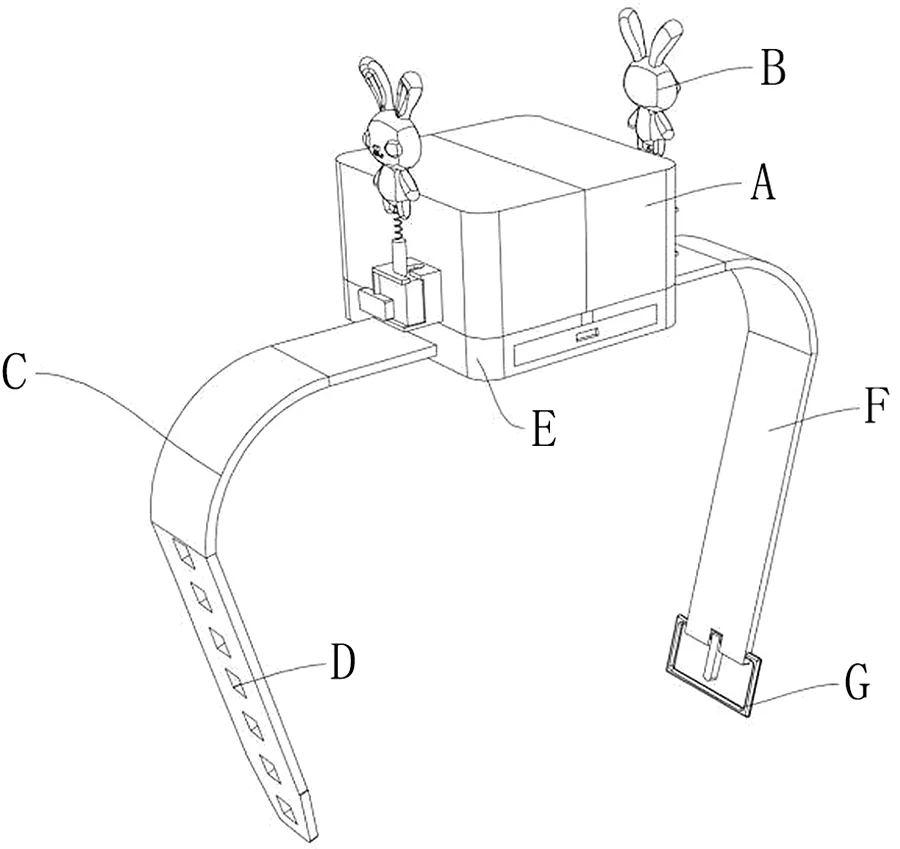
A novel intraoperative intravenous drug delivery device with independent intellectual property rights. **(A)** Cannula docking area; **(B)** Infusion set and auxiliary infusion tubing junction; **(C)** Three-way connector interface; **(D)** Drug administration tubing; **(E)** Cannula needle interface; **(F)** Infusion set interface; **(G)** Auxiliary infusion tubing; **(H)** Three-way connector port; **(I)** Robert clamp.

**Figure 2:**
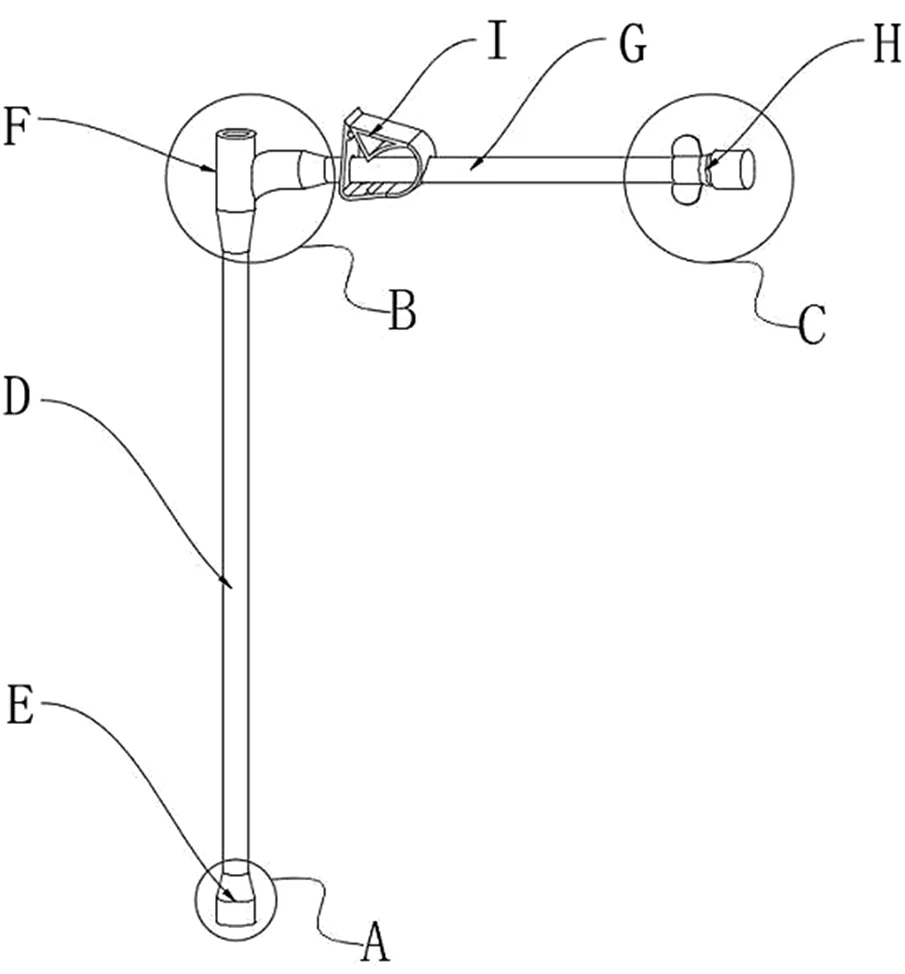
Adjustable peripheral intravenous catheter stabilizer. **(A)** Protective cover; **(B)** Central component; **(C)** First fastening strap; **(D)** Buckle hole; **(E)** Base plate; **(F)** Second fastening strap; **(G)** Buckle joint.

##### 2.2.4.4 Interdepartmental collaboration

The operating room head nurse coordinated with ward nursing leaders and the central transport team to standardize preoperative intravenous access preparation and fluid management during patient transfer.

##### 2.2.4.5 Audit and feedback

Daily case audits, monthly nursing reviews, and structured feedback were used to identify unresolved safety problems and incorporate corrective actions into subsequent PDCA cycles.

#### 2.2.5 Check (C): monitoring and feedback

The head nurse randomly audited daily cases to identify and report IV access issues. Feedback was promptly communicated to ward nursing staff to facilitate continuous improvement, ensuring safe and effective infusion management.

#### 2.2.6 Act (A): quality improvement and iteration

Daily summaries of IV-related issues were compiled by surgical nurses. Monthly nursing rounds were conducted to analyze and address these issues. Successful interventions were institutionalized, and unresolved problems were incorporated into the next PDCA cycle to ensure sustainable improvement in patient safety and care quality.

The process-management measures and the two auxiliary devices were introduced simultaneously as a bundled intervention, the study was designed to evaluate the overall strategy rather than the independent effect of each individual component. No factorial, sequential, or device-only comparison group was available to isolate component-specific effects.

### 2.3 Observation indicators

The primary outcome was catheter occlusion, defined as inability to infuse fluid through the catheter or loss of catheter patency despite routine repositioning and flushing attempts. Secondary outcomes included catheter-site erythema/tenderness, purulent discharge/blisters, thrombophlebitis, catheter dislodgement, peripheral leakage/infiltration, infusion-set dislodgement, dressing detachment, catheter dwell time, length of hospital stay, and patient satisfaction. Outcomes were assessed using predefined operational criteria. Local catheter-site infection/inflammation was defined by the presence of erythema, tenderness, induration, purulent drainage, or other clinically documented inflammatory signs at the PIVC insertion site. Phlebitis was defined as the presence of at least one clinically documented sign of venous inflammation, including localized warmth, tenderness or pain, erythema, swelling, or a palpable venous cord along the cannulated vein. Catheter occlusion was defined as inability to infuse fluid through the catheter or loss of catheter patency despite routine repositioning and flushing attempts. Peripheral leakage/infiltration was defined as unintended leakage of non-vesicant infused fluid into the surrounding tissue, accompanied by swelling, coolness, discomfort, or visible leakage at the insertion site. Catheter dislodgement was defined as partial or complete unintended withdrawal of the catheter from its original intravascular position. Infusion-set dislodgement was defined as unintended separation of any component of the infusion tubing system. Dressing detachment was defined as partial or complete loss of dressing adhesion requiring replacement or reinforcement. Catheter dwell time was calculated from catheter insertion to catheter removal, and length of hospital stay was calculated from admission to discharge.

Patient satisfaction with infusion-related nursing care was assessed using a locally developed 20-item questionnaire routinely administered as part of the hospital's discharge nursing quality-assessment program (Table 1). Each item was rated on a five-point Likert scale (1 = very dissatisfied to 5 = very satisfied), yielding a total score ranging from 20 to 100, with higher scores indicating greater satisfaction. For patients in the historical control cohort, satisfaction scores were not collected retrospectively after initiation of the present study; instead, they were extracted from contemporaneously completed discharge questionnaires archived in the hospital nursing quality-management records. The same questionnaire, scoring system, and administration procedure were used during both treatment periods.

**Table 1:** Items and scoring of the 20-item patient satisfaction questionnaire.

Category	Sub-items	Score Range
Nurse–Patient Communication	Introduction of surgical team and preoperative precautions	1–5 points
	Verification of bed number, patient name, and surgical site upon admission and entry to OR	1–5 points
	Introduction to operating room environment, staff, and safety information	1–5 points
	Psychological support and reassurance to reduce patient fear	1–5 points
Patient Health Education	Brief explanation of the disease and surgical procedure	1–5 points
	Explanation of the purpose of PIV catheter placement	1–5 points
	Instructions regarding catheter-related precautions	1–5 points
Puncture Technique	Satisfaction with puncture technique	1–5 points
	Success on first attempt	1–5 points
	Pain during puncture	1–5 points
	Skillfulness during the procedure	1–5 points
	Inquiry about patient's comfort during puncture	1–5 points
Nurse Attitude	Satisfaction with intraoperative positioning by nurses	1–5 points
	Return of personal belongings (e.g., clothes, medical records) to ward after surgery	1–5 points
	Attitude of OR nursing staff	1–5 points
	Satisfaction with OR environment	1–5 points
	Respect shown by nurses during the procedure	1–5 points
	Privacy protection	1–5 points
	Patience in answering questions	1–5 points
	Overall satisfaction with OR nursing care	1–5 points

Each item was scored from 1 (very dissatisfied) to 5 (very satisfied), yielding a total score of 20–100 points; higher scores indicated greater satisfaction. In the present cohort, Cronbach's *α* was 0.91, corrected item-total correlations ranged from 0.42 to 0.76, split-half reliability was 0.87, and the KMO value was 0.88.

The internal consistency and construct validity of the questionnaire were evaluated in the study sample. Cronbach's *α* for the overall 20-item scale was 0.91, indicating good internal consistency. Corrected item-total correlations ranged from 0.42 to 0.76, with all items exceeding the prespecified minimum acceptable value of 0.30. Split-half reliability was 0.87. Exploratory factor analysis showed adequate sampling suitability [Kaiser–Meyer–Olkin (KMO) measure = 0.88], and Bartlett's test of sphericity was significant (*χ*^2^ = 786.4, *P* < 0.001), supporting the factorability of the correlation matrix. Exploratory factor analysis identified four interpretable domains corresponding broadly to nurse–patient communication, health education, puncture-related care, and nursing attitude, which together explained 68.3% of the total variance.

### 2.4 Statistical analysis

All analyses were performed using SPSS version 26.0. Categorical variables were summarized as frequencies and percentages [n (%)] and compared using the chi-square test or Fisher's exact test, as appropriate. Normally distributed continuous variables were expressed as mean ± standard deviation and compared using the independent-samples *t*-test. Non-normally distributed continuous variables were summarized as median and interquartile range and compared using the Mann Whitney *U*-test. For dichotomous outcomes, effect estimates were expressed as risk ratios (RRs) with 95% confidence intervals (CIs), comparing the intervention cohort with the historical control cohort. For non-normally distributed continuous outcomes, between-group location shifts were additionally estimated using the Hodges Lehmann estimator with corresponding 95% CIs.

The psychometric properties of the locally developed satisfaction questionnaire were also assessed. Internal consistency was evaluated using Cronbach's *α* and corrected item-total correlations. Split-half reliability was assessed using the Spearman–Brown coefficient. Construct validity was explored using exploratory factor analysis. Sampling adequacy was evaluated using the Kaiser Meyer Olkin measure and Bartlett's test of sphericity. Factors with eigenvalues >1 were initially retained, and the factor structure was interpreted according to questionnaire domains and factor loadings.

The historical control and intervention cohorts were derived from different calendar periods, baseline demographic, clinical, and surgical characteristics were compared to assess potential temporal differences in case mix. Age, sex, and type of surgery were selected *a priori* as clinically relevant covariates. Multivariable logistic regression was performed for major binary outcomes with sufficient event numbers, with intervention period as the principal exposure and age, sex, and type of surgery entered as covariates. Adjusted associations were reported as adjusted odds ratios (aORs) with 95% CIs. Outcomes with very sparse event counts or marked imbalance between groups were not subjected to conventional multivariable logistic regression because of the risk of unstable estimates and model overfitting. Accordingly, catheter dislodgement, for which only one event occurred in the intervention cohort, was evaluated using the unadjusted risk ratio but was not included in the conventional multivariable logistic regression analysis. Adjusted analyses were considered supportive because of the modest sample size and historical-control design. All tests were two-sided, with *P* < 0.05 considered statistically significant.

## 3 Results

### 3.1 Comparison of baseline characteristics between the two groups

A total of 92 adult patients were included, comprising 46 patients in the historical control cohort and 46 patients in the intervention cohort. The historical control cohort included 30 men and 16 women, whereas the intervention cohort included 26 men and 20 women. The median ages were 54.5 years (IQR, 40.0–69.0) and 62.0 years (IQR, 51.75–67.0), respectively. No significant between-group differences were observed in age (*P* = 0.264), sex (*P* = 0.393), marital status, education level, clinical characteristics, or type of surgery (*P* = 0.476 for surgical procedure distribution; all *P* > 0.05). Detailed baseline demographic, clinical, and surgical characteristics are presented in Table 2.

**Table 2:** Baseline demographic, clinical, and surgical characteristics of the historical control and intervention cohorts.

Variable	Historical control (*n* = 46)	Intervention (*n* = 46)	Statistic	*P* value
Demographic characteristics				
Age, years, median (IQR)	54.50 (40.00–69.00)	62.00 (51.75–67.00)	Z = −1.117	0.264
Male sex, *n* (%)	30 (65.2)	26 (56.5)	*χ*^2^ = 0.730	0.393
Marital status, *n* (%)			2.687	0.507^a^
Unmarried	6 (13.0)	7 (15.2)		
Married	37 (80.4)	36 (78.3)		
Divorced	1 (2.2)	3 (6.5)		
Widowed	2 (4.3)	0 (0.0)		
Education level, *n* (%)			χ^2^ = 3.370	0.498
Primary school or below	12 (26.1)	12 (26.1)		
Junior high school	8 (17.4)	14 (30.4)		
Senior high school	8 (17.4)	8 (17.4)		
Associate degree	8 (17.4)	7 (15.2)		
Bachelor's degree or above	10 (21.7)	5 (10.9)		
Clinical characteristics				
Multidrug-resistant TB, *n* (%)	28 (60.9)	30 (65.2)	χ^2^ = 0.185	0.667
Diabetes mellitus, *n* (%)	8 (17.4)	10 (21.7)	χ^2^ = 0.274	0.601
Hypertension, *n* (%)	9 (19.6)	11 (23.9)	χ^2^ = 0.253	0.615
Chronic pulmonary comorbidity, *n* (%)	7 (15.2)	8 (17.4)	χ^2^ = 0.080	0.777
ASA physical status III, *n* (%)	14 (30.4)	16 (34.8)	χ^2^ = 0.200	0.655
Preoperative hemoglobin, g/L, mean ± SD	119.6 ± 15.8	121.3 ± 16.2	*t* = −0.509	0.612
Preoperative albumin, g/L, mean ± SD	36.8 ± 4.9	37.3 ± 5.1	t = −0.479	0.633
Surgical characteristics				
Type of surgery, *n* (%)			χ^2^ = 1.589	0.476
Thoracoscopic pleural biopsy	15 (32.6)	13 (28.3)		
Thoracoscopic decortication + empyema clearance + lung repair + closed drainage	19 (41.3)	16 (34.8)		
Chest wall TB lesion debridement + resection	12 (26.1)	18 (39.1)		
Operative duration, min, median (IQR)	128 (105–165)	124 (101–160)	Z = −0.412	0.68
Estimated blood loss, mL, median (IQR)	180 (100–300)	160 (100–280)	Z = −0.536	0.592
Intraoperative transfusion, *n* (%)	6 (13.0)	5 (10.9)	χ^2^ = 0.098	0.754
Postoperative ICU admission, *n* (%)	7 (15.2)	6 (13.0)	χ^2^ = 0.090	0.764
Postoperative pulmonary complications, *n* (%)	8 (17.4)	7 (15.2)	χ^2^ = 0.080	0.777
Postoperative wound complications, *n* (%)	3 (6.5)	2 (4.3)	Fisher's exact	1

Data are presented as *n* (%), mean ± SD, or median (IQR), as appropriate.

a Fisher's exact test was used when expected cell counts were <5.

ASA, American Society of Anesthesiologists; IQR, interquartile range; TB, tuberculosis.

**Table 3:** Comparison of local catheter-site inflammatory and infectious events between groups.

Outcome	Control (*n* = 46)	Intervention (*n* = 46)	RR (95% CI), intervention vs. control	*P* value
Erythema/tenderness	32 (69.6%)	4 (8.7%)	0.13 (0.05–0.33)	<0.001
Purulent discharge/blisters	8 (17.4%)	6 (13.0%)	0.75 (0.28–1.99)	0.772
Thrombophlebitis	5 (10.9%)	3 (6.5%)	0.60 (0.15–2.37)	0.711

RR, risk ratio; CI, confidence interval. RR < 1 favors the intervention cohort. Fisher's exact test was used when expected cell counts were <5.

### 3.2 Comparison of infusion-related infection rates

As shown in Table 3, compared with the historical control cohort, the intervention cohort had a substantially lower incidence of erythema and tenderness at the catheter site [8.7% vs. 69.6%; RR, 0.13; 95% CI, 0.05–0.33; *P* < 0.001]. No statistically significant differences were observed for purulent discharge/blister formation [13.0% vs. 17.4%; RR, 0.75; 95% CI, 0.28–1.99; *P* = 0.772] or thrombophlebitis [6.5% vs. 10.9%; RR, 0.60; 95% CI, 0.15–2.37; *P* = 0.711]. These findings represent associations with implementation of the overall intervention bundle and should not be interpreted as evidence of an independent effect of the PDCA framework or either auxiliary device.

### 3.3 Comparison of infusion-related safety issues between the two groups

As shown in Table 4, the intervention cohort had lower rates of catheter dislodgement [2.2% vs. 28.3%; RR, 0.08; 95% CI, 0.01–0.56; *P* = 0.001] and catheter occlusion [4.3% vs. 43.5%; RR, 0.10; 95% CI, 0.02–0.40; *P* < 0.001] than the historical control cohort. No statistically significant differences were observed for peripheral leakage [RR, 0.29; 95% CI, 0.06–1.30], infusion-set dislodgement [RR, 0.50; 95% CI, 0.13–1.88], or dressing detachment [RR, 0.75; 95% CI, 0.40–1.40].

**Table 4:** Comparison of infusion-related safety events between groups.

Outcome	Control (*n* = 46)	Intervention (*n* = 46)	RR (95% CI), intervention vs. control	*P* value
Peripheral leakage	7 (15.2%)	2 (4.3%)	**0.29** (**0.06–1.30)**	0.16
Infusion-set dislodgement	6 (13.0%)	3 (6.5%)	**0.50** (**0.13–1.88)**	0.483
Catheter dislodgement	13 (28.3%)	1 (2.2%)	**0.08** (**0.01–0.56)**	0.001
Catheter occlusion	20 (43.5%)	2 (4.3%)	**0.10** (**0.02–0.40)**	<0.001
Dressing detachment	16 (34.8%)	12 (26.1%)	**0.75** (**0.40–1.40)**	0.497

Fisher's exact test was used when expected frequency <5.

### 3.4 Comparison of indwelling time and length of hospital stay

Compared to the control group, the experimental group exhibited a significantly shorter length of hospital stay (11 days vs. 19 days, *P* < 0.001) and a longer intravenous catheter indwelling time (5 days vs. 3 days, *P* < 0.001), as detailed in Table 5. The intervention cohort had a longer catheter dwell time than the historical control cohort [median, 5.0 (IQR, 4.0–6.0) vs. 3.0 (IQR, 2.0–4.0) days; Hodges–Lehmann difference, +2.0 days; 95% CI, 1.0–2.0; *P* < 0.001]. The intervention cohort also had a shorter observed hospital stay [11.0 (IQR, 8.0–18.0) vs. 19.0 (IQR, 17.0–24.5) days; Hodges–Lehmann difference, −7.0 days; 95% CI, −10.0 to −5.0; *P* < 0.001] (Table 5). Because hospital stay may be influenced by disease severity, surgical complexity, postoperative complications, discharge practices, and other time-dependent factors, this difference should not be attributed solely to the infusion-management intervention.

**Table 5:** Comparison of catheter dwell time and length of hospital stay between groups.

Outcome	Historical control (*n* = 46), Median (IQR)	Intervention (*n* = 46), Median (IQR)	Hodges–Lehmann difference^a^ (95% CI)	*P* value
Catheter dwell time, days	3.00 (2.00–4.00)	5.00 (4.00–6.00)	**+2.00 (1.00–2.00)**	<0.001
Length of hospital stay, days	19.00 (17.00–24.50)	11.00 (8.00–18.00)	**−7.00 (−10.00 to −5.00)**	<0.001

Data are presented as median (IQR).

a The Hodges–Lehmann estimator represents the location shift between the intervention and historical control cohorts, expressed as intervention minus control, with corresponding 95% confidence intervals. Between-group comparisons were performed using the Mann–Whitney *U*-test.

CI, confidence interval; IQR, interquartile range.

### 3.5 Reliability and validity of the satisfaction questionnaire

The KMO measure was 0.88, and Bartlett's test of sphericity was statistically significant (*χ*^2^ = 786.4, *P* < 0.001), indicating that the data were suitable for exploratory factor analysis. Four clinically interpretable factors were identified, broadly corresponding to nurse–patient communication, patient health education, puncture-related care, and nursing attitude, and together accounted for 68.3% of the total variance.

### 3.6 Comparison of patient satisfaction with infusion management

The intervention cohort had a higher patient satisfaction score than the historical control cohort [median, 95.0 (IQR, 92.0–96.0) vs. 91.5 (IQR, 89.0–93.0); Hodges–Lehmann difference, +3.0 points; 95% CI, 2.0–4.0; *P* < 0.001] (Table 6). The same questionnaire and administration procedure were used during both study periods and the instrument demonstrated acceptable internal consistency and construct validity in the present sample, these results support a between-period difference in reported satisfaction. Nevertheless, given the non-randomized historical-control design, this difference should be interpreted as an association with the overall intervention period rather than a causal effect of any individual intervention component.

**Table 6:** Comparison of patient satisfaction scores between groups.

Outcome	Historical control (*n* = 46), Median (IQR)	Intervention (*n* = 46), Median (IQR)	Hodges–Lehmann difference^a^ (95% CI)	*P* value
Patient satisfaction score	91.50 (89.00–93.00)	95.00 (92.00–96.00)	+3.00 (2.00–4.00)	<0.001

Patient satisfaction was assessed using the locally developed 20-item questionnaire, with total scores ranging from 20 to 100; higher scores indicated greater satisfaction.

a The Hodges–Lehmann estimator represents the location shift between groups, expressed as intervention minus historical control, with a 95% confidence interval. The between-group comparison was performed using the Mann–Whitney *U*-test.

CI, confidence interval; IQR, interquartile range.

### 3.7 Adjusted analyses

After adjustment for age, sex, and type of surgery, implementation of the multicomponent intervention remained associated with a lower likelihood of catheter-site erythema/tenderness (aOR, 0.05; 95% CI, 0.01–0.18; *P* < 0.001) and catheter occlusion (aOR, 0.07; 95% CI, 0.02–0.32; *P* < 0.001) (Supplementary Table S1). Catheter dislodgement was not included in the conventional multivariable logistic regression analysis because only one event occurred in the intervention cohort, resulting in sparse data and potentially unstable adjusted estimates. Its between-group association was therefore reported using the unadjusted risk ratio. Overall, the adjusted findings were consistent with the primary analyses but should be interpreted cautiously given the modest sample size and non-randomized historical-control design.

## 4 Discussion

In this non-randomized historical-control study, the multicomponent PDCA-based infusion safety strategy was associated with fewer PIV-related safety events, longer catheter dwell time, and higher patient satisfaction than conventional care in the earlier period. The strategy included a structured quality-improvement framework, staff training, standardized clinical protocols, interdepartmental collaboration, audit and feedback, and two auxiliary devices. Because these elements were delivered together, the observed differences should be interpreted as associations with the overall intervention bundle, not as evidence of an independent effect of PDCA or either device.

The intervention combined process-level and device-level changes. Process-level components included staff training, standardized catheter and infusion procedures, interdepartmental coordination, routine auditing, feedback, and iterative improvement under PDCA. The two devices were introduced to support drug administration and catheter stabilization. These components may act through different mechanisms. Process changes may reduce practice variation and improve adherence to safety procedures, while the devices may provide mechanical support for catheter stabilization and standardized infusion handling. However, all components were introduced at the same time, so their independent or relative contributions cannot be determined. Factorial, stepwise, or device-only designs would be needed to isolate component-specific effects.

PDCA has been used in many healthcare quality-improvement settings, where it supports problem identification, implementation, monitoring, feedback, and iterative refinement (14–16). In this study, those principles were applied to PIV infusion safety through standardized procedures, staff training, catheter surveillance, and interdepartmental coordination. Contemporary vascular-access standards also emphasize appropriate device and site selection, secure catheter stabilization, regular assessment of catheter necessity and function, and early recognition of complications such as infiltration, phlebitis, occlusion, and dislodgement (10, 11, 17). These standards are more directly relevant to the present intervention than evidence from unrelated administrative or laboratory applications of PDCA. Interdepartmental collaboration and coordinated multidisciplinary management may also support more consistent vascular-access care and complication prevention (18). Future implementation should therefore strengthen multi-level coordination and use data-driven adjustments to sustain improvements in nursing quality and clinical management.

Targeted management and specialized training may improve staff knowledge and skills in vascular-access maintenance and complication prevention. Structured education, clear responsibilities, and standardized procedures may also support adherence to infusion-safety practices. However, nurse competency and work efficiency were not measured as outcomes in this study. These potential benefits should therefore be treated as possibilities, not demonstrated effects of the intervention.

The auxiliary devices were additional components of the overall strategy and may have contributed to the observed improvements. Their independent effects cannot be determined from this design. Securement and maintenance of peripheral intravenous catheters are clinically important because inadequate stabilization and suboptimal catheter management may contribute to dislodgement, infiltration, occlusion, phlebitis, and premature device failure (10, 19, 20). The adjustable PIVC stabilizer was intended to provide additional mechanical stabilization, and the intraoperative drug-delivery device was designed to support organized and standardized infusion handling. These intended functions align with contemporary vascular-access principles that emphasize appropriate device selection, securement, catheter patency, routine assessment, and prompt identification of device-related complications (10). Adult vascular-access studies also highlight the clinical importance of catheter-related complications and dwell-time considerations (21–23). Because the devices were implemented together with staff training, standardized protocols, interdepartmental collaboration, and PDCA-based quality improvement, their individual clinical effects cannot be isolated.

In summary, the multicomponent intervention combined PDCA-based process improvement, standardized vascular-access management, and auxiliary devices. Contemporary infusion-therapy standards support structured assessment, securement, maintenance, and early recognition of peripheral intravenous catheter complications (10). Adult clinical evidence confirms that infiltration, phlebitis, occlusion, dislodgement, and premature catheter failure remain important vascular-access problems (20, 21). The present findings support the feasibility of an integrated infusion-safety strategy, but they do not establish the independent effectiveness of PDCA or either device. Further prospective studies with concurrent controls and component-specific comparisons are needed before broader clinical implementation can be recommended.

This study has several limitations. First, it used a non-randomized historical-control design rather than a concurrent randomized comparison. The control cohort was treated between June 2023 and June 2024, and the intervention cohort was treated between July 2024 and April 2025. Although the same eligibility criteria were applied and measured baseline characteristics, including age, sex, and type of surgery, did not differ significantly between groups, temporal confounding cannot be excluded. Changes over time in staffing, clinical experience, surgical practice, infection-control procedures, perioperative care, discharge practices, or unmeasured patient characteristics may have contributed to the observed differences. The findings should therefore be interpreted as associations rather than definitive causal effects. Second, the intervention was implemented as an integrated bundle that included PDCA, staff training, standardized infusion protocols, interdepartmental collaboration, audit and feedback, and two auxiliary devices. Because these components were introduced simultaneously, their independent and relative contributions could not be separated. This study cannot determine whether the improvements were mainly due to process-management changes, the devices, or their combined implementation. Third, the sample size was relatively small, and the low number of events for some outcomes limited multivariable adjustment. Residual confounding may remain even after measured baseline characteristics are considered. Length of hospital stay is also influenced by factors beyond infusion management, including disease severity, surgical complexity, postoperative complications, and discharge criteria. Future prospective multicenter studies with concurrent controls, larger samples, and component-specific or factorial designs are needed to confirm these findings and clarify the contribution of individual components. Fourth, patient satisfaction was assessed with a locally developed 20-item questionnaire. The questionnaire showed good internal consistency and acceptable exploratory construct validity in this sample, but these psychometric analyses were conducted in the same relatively small single-center cohort. Independent external validation was not available. The generalizability of the satisfaction measure therefore remains uncertain. Future studies should validate the instrument in independent and multicenter populations and, where possible, compare it with established patient-reported satisfaction measures.

In conclusion, in this non-randomized historical-control study, the multicomponent PDCA-based infusion safety strategy was associated with fewer PIV-related safety events, longer catheter dwell time, and higher patient satisfaction than historical conventional care in adults undergoing thoracic surgery for pulmonary tuberculosis. The intervention included process-management measures such as staff training, standardized protocols, interdepartmental collaboration, audit, and feedback, together with two auxiliary devices. Because these components were implemented simultaneously and the comparison involved different treatment periods, the findings cannot establish an independent causal effect of PDCA or either device. Prospective studies with concurrent controls and component-specific comparisons are needed to confirm these associations.

## Data Availability

The raw data supporting the conclusions of this article will be made available by the authors, without undue reservation.

## Supplementary material

SUPPLEMENTARY TABLE S1 Unadjusted and Adjusted Associations Between the Multicomponent Intervention and Major PIV-Related Outcomes
