# Supplementary material for: Effectiveness of a multicomponent PDCA-based infusion safety management strategy in patients with pulmonary tuberculosis: a non-randomized historical-control study
Source: Front Med (Lausanne). 2026 Oct 6;13:1899920. doi: 10.3389/fmed.2026.1899920 (PMC13680403; doi:10.3389/fmed.2026.1899920)
Supplement: Supplementary file 1 [file Table1.docx]

Supplementary Table 1 Unadjusted and Adjusted Associations Between the Multicomponent Intervention and Major PIV-Related Outcomes

| **Outcome** | **Unadjusted RR (95% CI)** | **Adjusted OR (95% CI)*** | **P value** |
| --- | --- | --- | --- |
| Catheter-site erythema/tenderness | 0.13 (0.05–0.33) | 0.05 (0.01–0.18) | <0.001 |
| Catheter occlusion | 0.10 (0.02–0.40) | 0.07 (0.02–0.32) | <0.001 |
| Catheter dislodgement | 0.08 (0.01–0.56) | Not modeled† | 0.001‡ |

Note: *Adjusted for age, sex, and type of surgery. †Catheter dislodgement was not included in conventional multivariable logistic regression because only one event occurred in the intervention cohort, resulting in sparse data and potentially unstable adjusted estimates. ‡P value from the unadjusted between-group comparison. OR, odds ratio; RR, risk ratio; CI, confidence interval.
